# Orlistat Resensitizes Sorafenib-Resistance in Hepatocellular Carcinoma Cells through Modulating Metabolism

**DOI:** 10.3390/ijms23126501

**Published:** 2022-06-10

**Authors:** Pei-Wei Shueng, Hui-Wen Chan, Wei-Chan Lin, Deng-Yu Kuo, Hui-Yen Chuang

**Affiliations:** 1 Division of Radiation Oncology, Department of Radiology, Far Eastern Memorial Hospital, New Taipei City 220, Taiwan; shuengsir@gmail.com; 2 Faculty of Medicine, School of Medicine, National Yang Ming Chiao Tung University, Taipei City 112, Taiwan; 3 Department of Biomedical Imaging and Radiological Sciences, National Yang Ming Chiao Tung University, Taipei City 112, Taiwan; viator70757@gmail.com; 4 Department of Radiology, Cathay General Hospital, Taipei City 106, Taiwan; martinlin77@gmail.com; 5 School of Medicine, Fu-Jen Catholic University, New Taipei City 242, Taiwan

**Keywords:** sorafenib resistance, fatty acid synthase, metabolism, hepatocellular carcinoma

## Abstract

Sorafenib is one of the options for advanced hepatocellular carcinoma treatment and has been shown to extend median overall survival. However, sorafenib resistance often develops a few months after treatment. Hence, developing various strategies to overcome sorafenib resistance and understand the possible mechanisms is urgently needed. We first established sorafenib-resistant hepatocellular carcinoma (HCC) cells. Then, we found that sorafenib-resistant Huh7 cells (Huh7/SR) exhibit higher glucose uptakes and express elevated fatty acid synthesis and glucose metabolism-related proteins than their parental counterparts (Huh7). The current study investigated whether sorafenib resistance could be reversed by suppressing fatty acid synthesis, using a fatty acid synthase (FASN) inhibitor, orlistat, in HCC cells. FASN inhibition-caused changes in protein expressions and cell cycle distribution were analyzed by Western blot and flow cytometry, and changes in glucose uptakes were also evaluated by ^18^F-FDG uptake. Orlistat remarkably enhanced the cytotoxicity of sorafenib in both Huh7 and Huh7/SR cells, and flow cytometry showed that combination treatment significantly increased the sub-G1 population in both cell lines. Western blot revealed that the combination treatment effectively increased the ratio of Bax/Bcl-2 and decreased expressions of pERK; additionally, the combination treatment also strongly suppressed fatty acid synthesis-related proteins (e.g., FASN and SCD) in both cell lines. Lastly, the ^18^F-FDG uptake was repressed by the combination treatment in both cell lines. Our results indicated that orlistat-mediated FASN inhibition could overcome sorafenib resistance and enhance cell killing in HCC by changing cell metabolism.

## 1. Introduction

Hepatocellular carcinoma (HCC) is a commonly diagnosed cancer and ranks the ninth leading cause of cancer deaths worldwide [1]. Most HCC patients are diagnosed at an advanced stage, but surgical removal and embolization strategies are infeasible for treating advanced HCC. A multi-kinase inhibitor, sorafenib, serves as an option for patients with advanced HCC, when their conditions are allowed. However, sorafenib can only benefit around 1/3 of patients, only slightly improves patient survival, and HCC heterogeneity often results in irresponsiveness and an acquired resistance to sorafenib [2]. Different mechanisms related to sorafenib resistance have been proposed, from genetic and epigenetic regulation, cellular transport processes, cell death regulation, and tumor microenvironment modification [3]. We are interested in cellular metabolism, especially the fatty acid synthesis pathway, among these observations.

Lipids are involved in protein modification, steroid hormone synthesis, and the maintenance of cell membrane integrity. Similar to prostate cancer [4], HCC has been shown to overexpress types of lipogenic enzymes, including acetyl-CoA carboxylase (ACC), ATP citrate lyase (ACL), and fatty acid synthase (FASN). HCC cells also exhibit increased lipogenesis compared to surrounding hepatocytes [5], and enhanced lipogenesis strongly correlates to poor prognosis in HCC [6,7]. Several studies have reported that FASN expression positively correlates to cancer stages, including prostate cancer [8], renal cancer [9], and colorectal cancer [10]. Moreover, FASN inhibition can decrease the cancer cell stemness in breast cancer [11,12] and glioma [13]. Interestingly, it has been shown that sorafenib-resistant HCC cells demonstrate more characteristics of cancer stem cells than their parental counterparts [14,15].

Akt regulates the activity of sterol regulatory element-binding protein 1 (SREBP1), which controls FASN expression. Multiple cancer types, including HCC, show an activated Akt/mTOR pathway; nevertheless, the Akt is more upregulated in sorafenib-resistant HCC cells than their parental counterparts. Elevated mTOR promotes steatosis and tumorigenesis in hepatocytes by enhancing lipid synthesis [16,17]. mTOR is negatively regulated by AMPK, the critical metabolism modulator. FASN-overexpressed cells often show decreased AMPK, and FASN inhibition has been shown to re-activate AMPK in different cancer types [18,19]. AMPK activation could suppress cancer growth by inhibiting lipogenesis [20,21,22], and AMPK activators have been shown to suppress proliferation, invasion, and migration, like pancreatic cancer [23] and HCC [24,25]. Metformin, a first-line diabetes drug, can also activate AMPK and exhibit anti-cancer activity [26]. Metformin has been shown to sensitize HCC to sorafenib and decrease metastasis and recurrence after surgical removal in mice [27]. Moreover, a clinical report stated that a combination of metformin and sorafenib prolonged the survival of HCC patients [28].

Additionally, sorafenib results in cell death, mainly by inducing ferroptosis instead of apoptosis in several cancer cell lines [29,30]. Ferroptosis, an iron-dependent cell death, has been related to multiple diseases, including cancer [31], and was identified in 2012 by Dixon et al. [32]. Iron accumulation promotes Fenton reaction and lipid peroxidation, and finally leads to ferroptosis cell death. Lipid peroxidation preferentially oxidizes long-chain polyunsaturated fatty acids (PUFAs) derived from palmitate, and FASN regulates its synthesis. Interestingly, Liu and colleagues first demonstrated that sorafenib could disrupt stearoyl coenzyme A desaturase 1 (SCD1) and monounsaturated fatty acid (MUFA) production, and decrease ATP generation in liver cancer [33].

Based on the literature search results, we hypothesized that targeting FASN may re-sensitize HCC cells to sorafenib. Orlistat, a FASN inhibitor, has been shown to exert anti-tumor activity by several groups, including us [34,35,36,37,38]. We first compared the sensitivity to sorafenib in both parental and sorafenib-resistant HCC cells using the MTT assay. The cell growth curves, migration, protein expressions related to survival and lipogenesis, and to intracellular glycogen levels of both cell lines, were also analyzed. Then we combined orlistat with sorafenib to treat HCC cells, and studied how FASN inhibition affects sorafenib-resistant HCC cells from cell cycle distribution, protein expressions, and glucose uptake.

Here, we demonstrated that the expressions of FASN and other lipogenic enzymes are upregulated in sorafenib-resistant HCC cells. Orlistat significantly augmented the cytotoxicity of sorafenib in both parental and sorafenib-resistant HCC cells by enhancing apoptosis, and altering cell metabolism in sorafenib-resistant HCC cells was suppressed by the combination treatment. Our results indicated that FASN plays an essential role in developing sorafenib resistance in HCC by regulating cancer metabolism; thereby, FASN inhibition may be a possible means to improve the efficacy of sorafenib in HCC treatment.

## 2. Results

### 2.1. Huh7/SR Cells Show Higher Migratory Abilities than Parental Huh7 Cells

**Figure 1:**
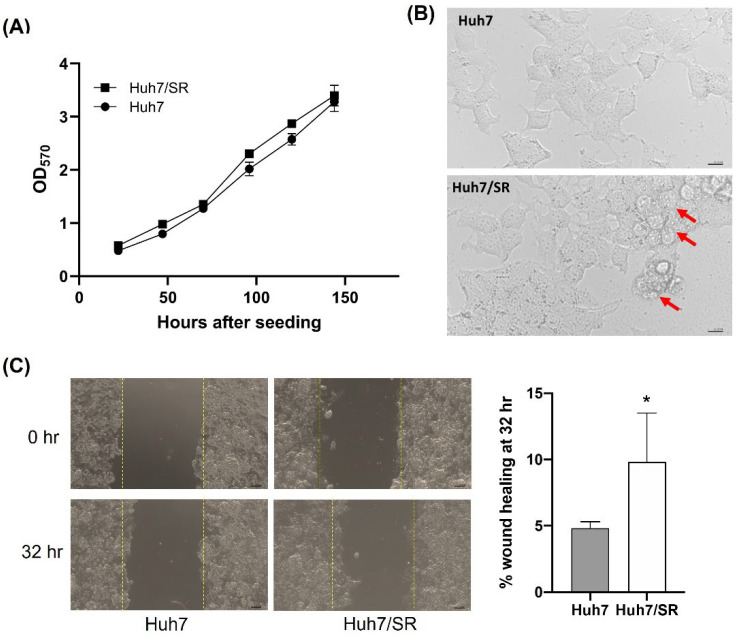
Comparison of cell growth rate and migratory ability between Huh7 and Huh7/SR cells. (**A**) Huh7 and Huh7/SR cells had similar growth rates, as shown by the MTT assay. (**B**) Cell morphology of Huh7 and Huh7/SR cells were observed by a light microscope. Red arrows point out the vesicle-like structures. (**C**) Wound healing assay was performed to determine the cellular migratory ability. Huh7/SR cells showed significantly higher migratory ability than Huh7 cells. * *p* < 0.05.

Growth curves of both Huh7 and Huh7/SR cells were drawn based on their MTT readouts to understand whether sorafenib resistance would accelerate cell proliferation in culture. Both cell lines showed similar plating efficiency and growth rates (Figure 1A), although Huh7/SR cells had slightly higher OD570 readings at 96- and 120-h time points. The morphology of Huh7 and Huh7/SR cells were examined using a light microscope, and some vesicle-like structures were noticed in Huh7/SR cells. Drug-resistant cells are often more invasive than their parental counterparts, and tend to form distal metastases. Therefore, the migratory abilities of both cell lines were examined by wound-healing assay. Huh7/SR cells showed a higher percentage of wound closure at the 32-h time point than Huh7 cells (Figure 1C), indicating that sorafenib resistance may enhance cell migration ability.

### 2.2. Protein Expression Profiles Are Different between Huh7 and Huh7/SR Cells

**Figure 2:**
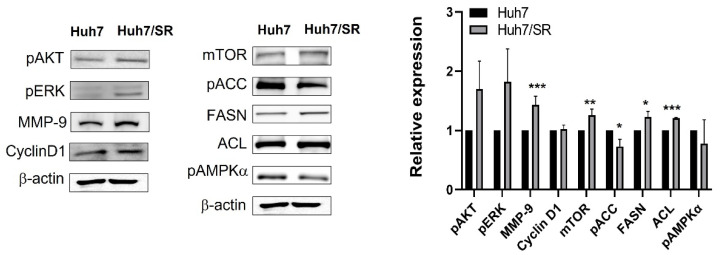
Huh7 and Huh7/SR cells show different protein expression profiles as determined by Western blot. Huh7/SR cells expressed higher pAKT, pERK, and MMP-9 related to cell survival and migration ability than Huh7 cells. Additionally, Huh7/SR cells showed elevated FASN, ACL, and mTOR correlated with fatty acid synthesis and metabolism. * *p* < 0.05; ** *p* < 0.01; *** *p* < 0.001.

Western blot was carried out to compare differences in protein expressions between Huh7 and Huh7/SR cells. Sorafenib suppresses HCC growth through ERK inhibition and could induce Akt activation; hence, pAkt and pERK levels were determined in both cell lines. Figure 2 shows that Huh7/SR cells had higher pAkt and pERK expressions than Huh7 cells. The results obtained from growth curves and wound-healing assays indicated that Huh7/SR cells showed a comparable growth rate but a higher migratory ability than Huh7 cells. Next, we evaluated cyclin D1 and MMP-9 expressions in both cell lines and found that Huh7/SR cells expressed comparable cyclin D1 but higher MMP-9 than Huh7 cells.

The Akt and ERK pathways also modulate cell metabolism, except for triggering cell growth and migration. Therefore, several metabolism-related protein expressions in Huh7 and Huh7/SR cells were examined. Figure 2 exhibits that Huh7/SR cells expressed higher mTOR and lower AMPKα related to glucose metabolism regulation. Besides, fatty acid synthesis-related proteins including ACL and FASN were all upregulated in Huh7/SR cells. However, no significant difference in these protein expressions was found between Huh7 and Huh7/SR cells.

### 2.3. Orlistat Enhances Cytotoxicity of Sorafenib in Both Huh7 and Huh7-SR Cells

**Figure 3:**
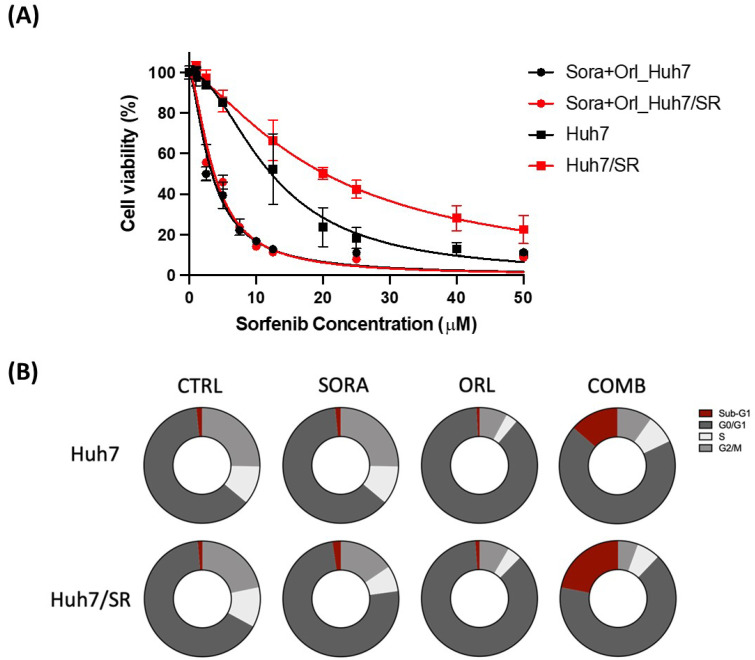
Orlistat combined with sorafenib significantly increased cell killing effects on Huh7 and Huh7/SR cells. (**A**) Cell viability of both cell lines was assessed by MTT assay after being treated with sorafenib combined with or without 50 µM orlistat. Huh7/SR cells had higher cell viability than Huh7 cells when treated with sorafenib alone (>10 µM). However, the combination treatment resulted in comparable cell killing in both cell lines. (**B**) Cell cycle distribution was evaluated by flow cytometry. Combination treatment remarkably increased the sub-G1 population in both cell lines, and the effect was more evident in Huh7/SR cells than in Huh7 cells. Besides, combination treatment caused a more significant G2/M reduction in Huh7/SR cells than in Huh7 cells.

Western blot showed that sorafenib-resistant Huh7/SR cells had higher expressions of several fatty acid synthesis-related proteins than Huh7 cells. Therefore, we hypothesized that inhibiting fatty acid synthesis may reverse the acquired sorafenib resistance. We have tested that 50 µM orlistat would cause < 15% of cell death in both cell lines (Figure S1), and chose this concentration of orlistat to be combined with sorafenib treatment. Figure 3A shows that 50 µM orlistat significantly enhanced sorafenib-mediated cell killing in both cell lines, although sorafenib was more effective at cell killing in Huh7/SR cells than Huh7 cells.

Flow cytometry was conducted to investigate how these treatments would influence the cell cycle progression. Results showed that the combination treatment strikingly increased the sub-G1 population in both cell lines, and 5 µM sorafenib or 50 µM orlistat alone did not cause notable cell death (Figure 3B). Orlistat resulted in more obvious G0/G1 arrest than sorafenib, and the combination treatment substantially reduced the S and G2/M populations. Table 1 shows the overall cell cycle distribution of cells that received various treatments.

### 2.4. Combination Treatment Changes Protein Expressions Differently in Huh7 and Huh7/SR Cells

**Figure 4:**
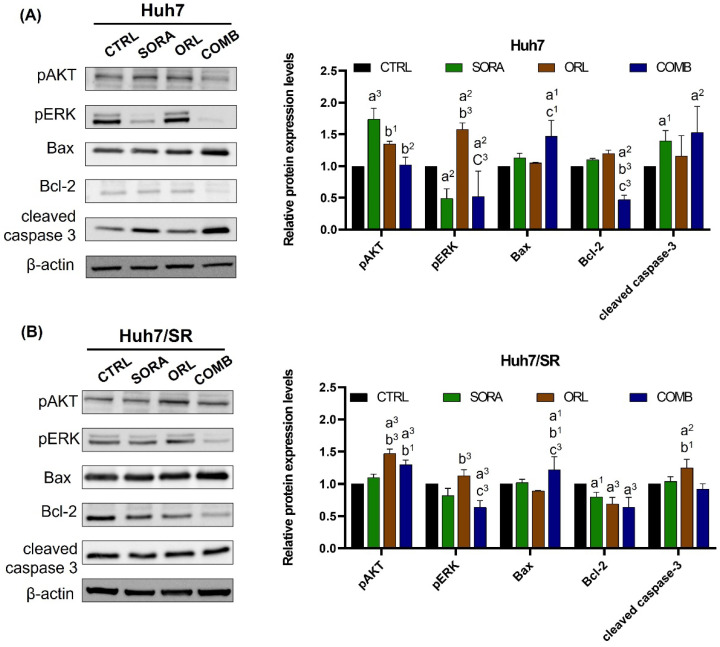
Effects of combination treatment of sorafenib and orlistat on proliferation and apoptosis pathways in (**A**) Huh7 and (**B**) Huh7/SR cells. Combination treatment effectively suppressed pERK and Bcl-2 expressions in Huh7 cells compared to single treatments. Decreased expression resulting from combination treatment was also seen in Huh7/SR cells but less pronounced. Additionally, combination treatment enhanced the BAX/Bcl-2 ratio in both cell lines. a, compared to Control; b, compared to Sorafenib; c, compared to Orlistat; ^1^ *p* < 0.05; ^2^ *p* < 0.01; ^3^ *p* < 0.001.

**Figure 5:**
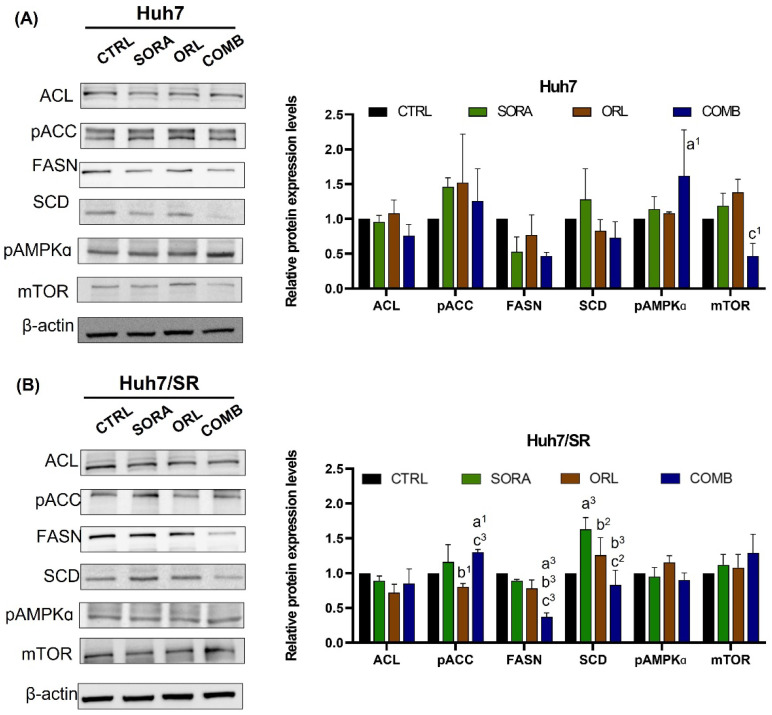
Combination treatment of sorafenib and orlistat changes the expressions of fatty acid synthesis-related and AMPK/mTOR-related proteins in (**A**) Huh7 and (**B**) Huh7/SR cells. Combination treatment caused reductions in FASN and SCD and increased pACC expression in both cell lines. Besides, the combination treatment activated the AMPK/mTOR pathway only in Huh7 cells but not in Huh7/SR cells. a, compared to Control; b, compared to Sorafenib; c, compared to Orlistat; ^1^ *p* < 0.05; ^2^ *p* < 0.01; ^3^ *p* < 0.001.

Sorafenib and orlistat are known to block the ERK and FASN pathways, respectively. Based on pERK and FASN expression changes, Huh7/SR cells were less sensitive to sorafenib and orlistat than Huh7 cells (Figure 4 and Figure 5). However, the combination treatment effectively suppressed pERK and FASN expressions in both cell lines compared to single treatments, especially in Huh7/SR cells. Worth noting that sorafenib and orlistat enhanced pAkt expression in Huh7 and Huh7/SR cells, respectively, indicating the inherent differences between these two cell lines. In addition, combination treatment increased Bax and decreased Bcl-2 levels in both cell lines; therefore, the Bax/Bcl-2 ratio increased and implied a potential apoptosis enhancement. However, combination treatment did not cause changes in cleaved caspase-3 in Huh7/SR cells as it did in Huh7 cells (Figure 4).

Figure 5 demonstrates that combination treatment led to more marked pACC elevation and FASN reduction, but a less-apparent change in ACL in both cell lines. Sorafenib increased the SCD expression in both cell lines, as shown in Figure 5. Still, the combination treatment reversed the sorafenib- and orlistat-mediated SCD elevation in both cell lines, especially in Huh7/SR cells. Besides, combination treatment elevated pAMPK expression in Huh7 cells, not observed in Huh7/SR cells.

**Figure 6:**
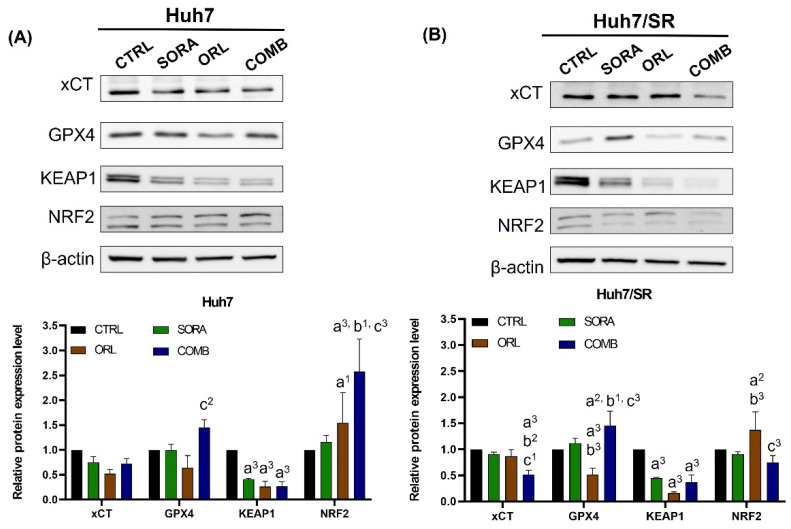
Combination treatment of sorafenib and orlistat altered expressions of ferroptosis-related proteins in Huh7 and Huh7/SR cells. Protein expression changes were detected by Western blot in (**A**) Huh7 and (**B**) Huh7/SR cells after treatments, which caused similar changes in xCT, GPX4, and KEAP1 expressions but had different effects on NRF2 expression in both cell lines. Significantly, orlistat and combination treatment decreased KEAP1 levels in both cell lines; both treatments only elevated NRF2 level in Huh7 cells but had little effect on Huh7/SR cells. a, compared to Control; b, compared to Sorafenib; c, compared to Orlistat; ^1^ *p* < 0.05; ^2^ *p* < 0.01; ^3^ *p* < 0.001.

As mentioned, sorafenib causes cell death mainly through ferroptosis instead of apoptosis. Figure 4 demonstrates that combination treatment increased the cleaved caspase-3 and Bax/Bcl-2 ratio more significantly in Huh7 cells than in Huh7/SR cells. Therefore, we investigated the changes in ferroptosis proteins resulting from treatments (Figure 6). Both sorafenib and orlistat suppressed xCT levels in both cell lines, and the suppression was less significant in Huh7/SR cells than in Huh7 cells. However, the reduction in xCT in Huh7/SR cells was more profound than in Huh7 cells after receiving combination treatment. All treatments had a similar effect on GPX4 expression, but the effect on Huh7/SR cells was less evident than on Huh7 cells. Lastly, all treatments repressed KEAP1 expression significantly, as shown in Figure 6. Nevertheless, treatments resulted in different changes in NRF2 expressions in both cell lines. Prominent NRF2 elevation was found in all treatment groups in Huh7 cells, but the NRF2 increase was only detected in Huh7/SR cells after orlistat treatment.

**Figure 7:**
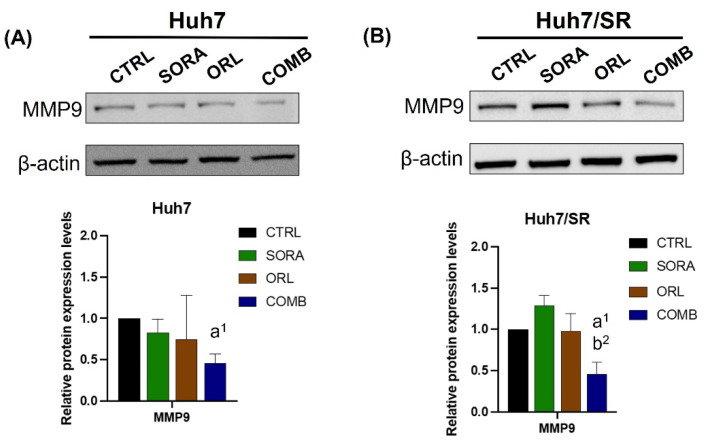
Combination treatment of sorafenib and orlistat repressed the MMP-9 expression in both (**A**) Huh7 and (**B**) Huh7/SR cells. Combination treatment suppressed MMP-9 level in both cell lines. a, compared to Control; b, compared to Sorafenib; ^1^ *p* < 0.05; ^2^ *p* < 0.01.

Figure 7 shows the changes in MMP-9 expression in Huh7 and Huh7/SR cells after receiving different treatments. Sorafenib elevated the MMP-9 expression only in Huh7/SR but not Huh7 cells, which echoed the findings observed in the wound-healing assay. Moreover, the MMP-9 expressions were effectively suppressed by the combination, implying that the combination treatment might decrease the invasiveness of both cell lines.

### 2.5. Combination Treatment Represses ^18^F-FDG Uptake in Huh7 and Huh7/SR Cells

**Figure 8:**
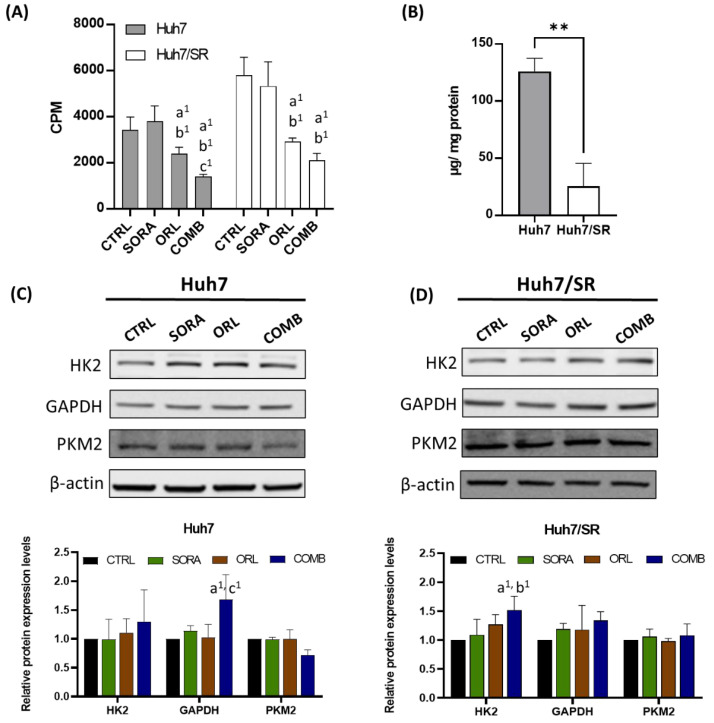
Combination treatment of sorafenib and orlistat diminished the elevated 18F-FDG uptakes detected in Huh7/SR cells and altered the expressions of glycolysis-related proteins. (**A**) Equal numbers of cells were cultured in the medium containing ^18^F-FDG for an hour after receiving different treatments. Sorafenib treatment did not change the ^18^F-FDG uptakes in both cell lines. However, the ^18^F-FDG uptakes were suppressed by orlistat and combination treatment, and the reduction percentages were more prominent in Huh7/SR cells. (**B**) Huh7 cells had higher intracellular glycogen than Huh7/SR cells, indicating that excess glucose uptakes in Huh7/SR cells were utilized for energy production rather than storage. Changes in HK2, GAPDH, and PKM2 expressions caused by treatments in (**C**) Huh7 and (**D**) Huh7/SR cells were determined by Western blot. a, compared to Control; b, compared to Sorafenib; c, compared to Orlistat; ^1^ *p* < 0.05; ** *p* < 0.01 between two groups.

Huh7 and Huh7/SR cells were incubated in a medium containing ^18^F-FDG (specific activity = 1 µCi/mL) for an hour to evaluate how different drug treatments affect their glucose uptakes. Huh7/SR cells had higher ^18^F-FDG uptakes than Huh7 cells under all conditions. Sorafenib did not decrease ^18^F-FDG uptake as orlistat did; additionally, the most significant ^18^F-FDG uptake suppression was observed in the cells receiving the combination treatment in both cell lines (Figure 8A).

An ^18^F-FDG uptake assay showed that Huh7/SR cells took up more glucose than Huh7 cells. We then aimed to understand why Huh7/SR cells showed a higher uptake, and measured the intracellular glycogen level in both cell lines. Figure 8B shows that Huh7 cells had higher glycogen concentration than Huh7/SR cells, indicating that Huh7/SR cells might use more glucose for generating ATP and producing materials for fast cell divisions. Besides, Western blot revealed that combination treatment increased the expressions of HK2 and GAPDH in both cell lines. However, the PKM2 changes in both cell lines were insignificant. (Figure 8C,D).

## 3. Discussion

Sorafenib has been the first-line treatment for patients with advanced HCC since 2007 [2,39]. Nevertheless, the acquired resistance causes sorafenib to only extend patients’ survival by 3.8 months [40] and limits treatment options available for patients with sorafenib resistance. Hence, new strategies that could overcome sorafenib resistance are needed to improve the prognosis of advanced HCC. The development of sorafenib resistance is related to genetic and epigenetic regulation, cellular metabolism, and cell death regulation [3]. Here, we aimed to investigate the roles of FASN and related metabolisms in sorafenib resistance, and to seek possible solutions to this pressing clinical issue.

We found that sorafenib-resistant Huh7/SR cells had a similar growth rate but higher migratory ability than Huh7 cells (Figure 1), which echoed their unchanged Cyclin D1 and increased MMP-9 expressions in Huh7/SR cells (Figure 2). Drug-resistant cells usually exert a higher migration tendency and aggressiveness leading to distal metastases [41,42,43]. Besides, metabolic changes may contribute to sorafenib resistance in HCC [44,45]. Huh7/SR cells expressed increased FASN, ACL, and mTOR expression compared to Huh7 cells, along with decreased pAMPKα (Figure 2). Orlistat significantly enhanced the cytotoxicity of sorafenib in both Huh7 and Huh7/SR cells, especially when the sorafenib dose was higher than 10 µM (Figure 3A). It has been reported that orlistat-mediated FASN inhibition reversed the resistance to taxane [46] and cisplatin [47] in prostate and ovarian cancer, respectively. Orlistat-related cell-killing augmentation has been seen when combined with traditional chemotherapeutic drugs, BRAF inhibitor [48], and proteasome inhibitor [49]. Flow cytometry results indicated that neither sorafenib nor orlistat affected the cell cycle distributions; however, combination treatment increased the sub-G1 population more significantly in Huh7/SR cells than in Huh7 cells (Figure 3B and Table 1).

Although treatments resulted in different changes in Akt, ERK, and cleaved caspase-3 expressions in Huh7 and Huh7/SR cells, combination treatment promoted pAkt but repressed pERK in both cell lines (Figure 4). The combination treatment led to substantial sub-G1 populations in both cell lines; however, only Huh7 cells had increased cleaved caspase-3 expression after receiving sorafenib and combination treatments. In contrast, combination treatment elevated Bax and reduced Bcl-2 expressions, increasing the ratio of Bax/Bcl-2 in both cell lines. The ratio of Bax/Bcl-2 could be used as an apoptotic index to predict the cell fate after treatment [50,51]. Balusamy et al. reported that citral suppressed tumor growth by inhibiting the fatty acid synthesis and inducing apoptosis through AMPK activation. Their study also reported increased Bax and decreased Bcl-2 levels after treatment in prostate cancer cells [52].

The combination treatment reduced FASN and SCD expressions but had opposite effects on pAMPKa and mTOR in both cell lines (Figure 5). Similarly, Liu and colleagues have reported that sorafenib could cause downregulation of SCD and increased AMPK phosphorylation in HCC [33]. Sorafenib could also activate AMPK and alter glucose metabolism in breast cancer cells [53]. FASN positively upregulated the AMPK/mTOR pathway and enhanced proliferation in colorectal cancer cells [54]. AMPK is a metabolic stress sensor regulated by oxygen concentration [55] and nutrient status [56]. Similar to our observations, FASN expression is also related to the survival and drug resistance in diffuse larger B-cell lymphoma [57]. Lipogenesis inhibition and fatty acid oxidation enhancement are correlated with upregulated AMPK and ACC phosphorylation [58,59], which were also observed in the cells receiving combination treatment in the current study. Phosphorylation of AMPK is known to suppress lipogenesis by downregulating FASN and SCD expressions; moreover, pAMPK enhances phosphorylation of ACC and represses the activity of ACC. As a result, fatty acid oxidation (FAO) will be inhibited because of the decreased malonyl-CoA and long-chain fatty acids. Inhibiting FAO has been shown to increase the Bax/Bcl-2 ratio and trigger apoptosis in leukemia [60] and glioblastoma [61].

Sorafenib has been shown to reduce the synthesis of polyunsaturated fatty acids (PUFAs) by inhibiting SCD1 expression [33]. Oxidation of PUFAs is highly related to lipid peroxidation seen in ferroptosis. Several studies have shown that sorafenib triggers ferroptosis by inhibiting xCT, affecting glutathione peroxidase, and then increasing ROS levels in HCC cells [29,30,62]. Figure 6 shows that sorafenib-mediated xCT/SLC7A11 reduction is less significant in Huh7/SR cells than in Huh7 cells. This finding is similar to recent studies showing that sorafenib-resistant HCC cells had higher xCT expression and compromised ferroptosis than their parental counterparts [63,64]. After receiving sorafenib treatment, there was a substantial KEAP1 reduction, and unchanged NRF2 expression was detected in Huh7 and Huh7/SR cells. Although the KEAP1 was suppressed in both cell lines after combination treatment, the opposite effect was observed on NRF2 expression. Activation of the p62-Keap1-NRF2 pathway has been shown to protect HCC cells against ferroptosis [65], leading to the development of sorafenib resistance [66]. Orlistat alone caused reductions in xCT and GPX4, indicating that orlistat might be a potential ferroptosis inducer. Nevertheless, orlistat also reduced KEAP1 and slightly increased NRF2 expression in both cell lines. Biosynthesis of PUFAs has been shown to determine the sensitivity to ferroptosis in gastric cancer cells [67]. Orlistat is a FASN inhibitor suppressing palmitic acid synthesis, which may also affect the activation of ferroptosis. Currently, only one report shows that orlistat could induce ferroptosis-like cell death in lung cancer [68].

Cells need to seek other energy supplies such as increasing glycolysis to survive to cover the energy shortage. Hence, we measured the ^18^F-FDG uptakes in cells receiving various treatments to see whether they upregulated glucose metabolism while their FASN was inhibited. Huh7/SR cells had significantly higher ^18^F-FDG uptakes than Huh7 cells under all conditions (Figure 8A), which resonated with lower pAMPK and higher mTOR expressions in Huh7/SR cells (Figure 2). We also found that Huh7 cells had higher intracellular glycogen levels than Huh7/SR cells (Figure 8B). The result seemed to conflict with ^18^F-FDG uptake and Western blot results since the Akt/mTOR pathway was upregulated in Huh7/SR cells. Akt is known to suppress glycogen synthase kinase-3 (GSK-3) and enhance glycogen synthesis [69]. HK2, GAPDH, and PKM2 are the glycolytic enzymes, and elevated glycolysis is related to tumor progression and treatment resistance [70]. We found that the expressions of HK2 and GAPDH were increased, and PKM2 was not affected after receiving the combination treatment in both cell lines (Figure 8C,D). These results implied that the remaining viable cells might exert resistance as expected, and glycolysis inhibition may be applied to eradicate the remaining cancer cells. Several inhibitors targeting glycolytic enzymes have been developed [71], and dual targeting of glycolysis and fatty acid oxidation has been shown to be effective and safe in glioblastoma treatment [61].

In conclusion, abnormal metabolism strongly correlates to the development of sorafenib resistance, which impairs the efficacy of sorafenib treatment in advanced HCC. We demonstrated that FASN inhibition might resensitize HCC cells to sorafenib by shifting metabolism and triggering apoptosis, and may play some role in regulating ferroptosis cell death. How orlistat or FASN inhibition interacts with sorafenib-induced ferroptosis requires further studies in the future. Additionally, the interactions resulting from the combination of orlistat or any FASN inhibition strategy with sorafenib need to be studied carefully to ensure its safety and efficacy for further application in advanced HCC in the future.

## 4. Materials and Methods

### 4.1. Cell Lines

The human hepatocellular carcinoma cell line, Huh7, was obtained from Dr. Jason Chia-Hsieh Cheng (National Taiwan University, Taipei, Taiwan) and utilized in this study. The establishment of a sorafenib-resistant Huh7 cell line (named Huh7/SR) was conducted by incubating Huh7 cells with medium containing 5 µM sorafenib for 21 consecutive days. Medium containing sorafenib was replaced every three days and the cells were split when they reached 80% confluency. After 21-day incubation in medium containing 5 μM sorafenib, the cells were named as Huh7/SR cells and used in the following experiments. Both Huh7 and Huh7/SR cells were maintained in DMEM supplemented with 10% fetal bovine serum (FBS, Corning, NY, USA) and 1% P/S (Hyclone, Logan, UT, USA), and kept in the 37 °C humidified CO_2_ incubator.

### 4.2. Drugs

Sorafenib (#10009644) and orlistat (#10005426) were purchased from Cayman Chemical (Ann Arbor, MI, USA). Sorafenib and orlistat were dissolved in DMSO and absolute ethanol, respectively. The stock solutions of sorafenib (40 mM) and orlistat (20 mM) were stored at −20 °C for the following experiments.

### 4.3. Cell Viability Assay

Huh7 or Huh7/SR cells were seeded in 96-well plates at the density of 1.5 × 10^4^ cells/well a day before drug treatments. Cells were then treated with various concentrations of sorafenib and/or orlistat for 48 h. The vehicle control groups were treated with 0.1% DMSO and/or 1% ethanol to help elucidate the cytotoxic effects of the treatments. Cell viability was assessed by MTT (3-(4,5-Dimethylthiazol-2-yl)-2,5-diphenyltetrazolium bromide)) assay. Briefly, 1/10 of the total volume of stock MTT solution (5 mg/mL) was added to each well (i.e., 10 µL MTT per 100 µL medium) and incubated for another 3 h. The formazan was dissolved with DMSO, and the absorbance at 570 nm was read by an ELISA reader (Tecan, Grödig, Austria).

### 4.4. Wound Healing

5 × 10^4^ Huh7 and Huh7/SR cells were seeded into the culture-insert (ibidi culture-insert 2 well, ibidi GmbH, Munich, Germany). Overnight incubation allowed cells to attach, then the culture-insert was taken off, and PBS was applied to remove the unattached cells, followed by fresh medium replacement. Cell migration was monitored for 32 h, and the pictures of the culture-insert were taken at different time points under a light microscope at 40× magnification. Image J software was used to quantify the percent of wound healing by comparing the images obtained at 0- and 32-h time points.

### 4.5. Flow Cytometry

Forty-eight hours after treatments (vehicles, 5 μM sorafenib, 50 μM orlistat, or combination treatment), Huh7 and Huh7/SR cells were collected, centrifuged at 1000× g for 10 min, and fixed with ice-cold 70% ethanol for at least 24 h. Cells were washed with PBS twice before being resuspended in staining solution (recipe: 100 µg/mL propidium iodide, 20 µg/mL RNaseA) and incubated in the dark at 37° for 30 min. Samples were analyzed by flow cytometry (Beckman Coulter CytoFLEX, Brea, CA, USA).

### 4.6. Western Blot

Huh7 or Huh7/SR cells were harvested and lysed with RIPA buffer (Visual protein, Taipei, Taiwan) containing Halt™ Phosphatase Inhibitor Cocktail (1:100 dilution, Thermofisher Scientific, Hanover Park, IL, USA) after being treated with vehicle: 5 μM sorafenib, 50 μM orlistat, or combination treatment for 48 h. Protein concentrations were determined by the Bradford assay, and 30–40 µg protein lysates were loaded in each lane and separated by electrophoresis using 8–12% SDS-PAGE gels. Proteins were transferred to a nitrocellulose membrane and blocked with 5% non-fat milk or 5% bovine serum albumin for an hour at room temperature to avoid nonspecific binding. Membranes were incubated with primary antibodies overnight at 4 °C and were further reacted with designated secondary antibodies (Genetex, Irvine, CA, USA) for an hour at room temperature. Signals were detected using the ECL Substrate (Visual protein) and acquired by a luminescence imaging system (LAS-4000, GE). Primary antibodies used were shown below: phosphor-AKT (Cell Signaling, Beverly, MA, USA), phosphor-ERK (Cell Signaling), Bax (Arigo, Hsinchu City, Taiwan), Bcl-2 (Arigo), Cleaved caspase-3 (Arigo), MMP-9 (Invitrogen, Waltham, CA, USA), CyclinD1 (Cell Signaling), mTOR (Genetex), phosphor-ACC (Cell Signaling), FASN (Genetex), ACL (Cell Signaling), SCD (Genetex), phospho-AMPKα (Cell Signaling), xCT/SLC7A11 (Cell Signaling), GPX4 (Cell Signaling), KEAP1 (Cell Signaling), and NRF2 (Cell Signaling). All primary antibodies were used at 1:1000 dilution, except for Bax, which was diluted at 1:2000.

The band intensities were quantified using ImageJ (National Institutes of Health, Bethesda, MD, USA). β-actin (Genetex) was used as an internal control. All the protein expressions were first normalized with their corresponding β-actin expression, and relative protein expressions were obtained by comparing the expressions in Huh7/SR cells to that in Huh7 cells.

### 4.7. ^18^F-FDG Uptake

Huh7 and Huh7/SR cells were seeded in a 24-well plate at the density of 5 × 10^5^ cells per well after receiving different treatments (vehicle, 5 μM sorafenib, 50 μM orlistat, and combination treatment) for 24 h. After cells were attached, 1 µCi 2-deoxy-2-(18F)fluoro-D-glucose, ^18^F-FDG (provided by The National PET/Cyclotron Center, Taipei, Taiwan) was added to each well (final specific activity = 1 µCi/mL). ^18^F-FDG is a radioactive glucose analogue used as a tracer. Both medium and cells were collected after one-hour incubation at 37 °C. Medium and cells were counted separately using a gamma counter (Wizard2 Gamma Counter, PerkinElmer, Cambridge, MA, USA).

### 4.8. Intracellular Glucogen Measurement

The intracellular glycogen levels in Huh7 and Huh7/SR cells were determined by the glycogen assay kit (Abcam, Cat. Ab65620, Cambridge, MA, USA) following the manufacturer’s protocol. Cells were homogenized in ddH2O on ice for 10 min, and then the samples were boiled for 10 min to inactivate enzymes. Supernatants were collected after 13,000 rpm centrifugation. The protein concentrations were measured using the Bradford assay, and 20–50 µg total protein with a final volume of 50 µL was added to each well of a 96-well plate. 2 µL of hydrolysis enzyme mix was first added to each well and incubated at room temperature for 30 min. 48 µL of reaction mix (containing developing buffer, developing enzyme, and probe) was then added, and the plate was read at OD450 nm using an ELISA reader after a 30-min incubation.

### 4.9. Statistical Analysis

All experiments were performed at least three times and the results are shown as mean ± standard deviation. Statistical analysis was performed using GraphPad Prism 9 software (GraphPad Software Inc.; San Diego, CA, USA). The student’s *t*-test was used when comparing two groups, and one-way ANOVA was used for multiple comparisons. A value of *p* < 0.05 was viewed as statistically significant.

## Figures and Tables

**Table 1:** The percentages of cells in each cell cycle phase in both Huh7 and Huh7/SR cells.

		Sub-G1	G0/G1	S	G2/M
Huh7	CTRL	1.50 ± 0.12	61.24 ± 1.76	10.58 ± 0.96	24.81 ± 1.30
	SORA	2.80 ± 0.89	67.01 ± 3.24	8.06 ± 1.72	21.64 ± 2.26
	ORL	0.84 ± 0.07	86.94 ± 1.70	3.22 ± 1.55	7.74 ± 1.41
	COMB	13.30 ± 6.81	66.86 ± 6.19	7.92 ± 2.64	9.61 ± 2.59
Huh7/SR	CTRL	1.26 ± 0.28	65.87 ± 5.21	11.22 ± 1.45	21.76 ± 5.38
	SORA	2.33 ± 1.90	73.98 ± 4.34	7.17 ± 1.20	15.39 ± 2.51
	ORL	1.16 ± 0.33	85.40 ± 6.14	3.96 ± 3.38	8.29 ± 1.78
	COMB	21.43 ± 7.51	64.51 ± 4.83	6.39 ± 0.62	5.46 ± 2.51

## Data Availability

Data is contained within the article or Supplementary Material.

## Supplementary Materials

The following supporting information can be downloaded at: https://www.mdpi.com/article/10.3390/ijms23126501/s1.
