# Supplementary figures and images for: Orlistat Resensitizes Sorafenib-Resistance in Hepatocellular Carcinoma Cells through Modulating Metabolism
Source: Int J Mol Sci. 2022 Jun 10;23(12):6501. doi: 10.3390/ijms23126501 (PMC9223797; doi:10.3390/ijms23126501)

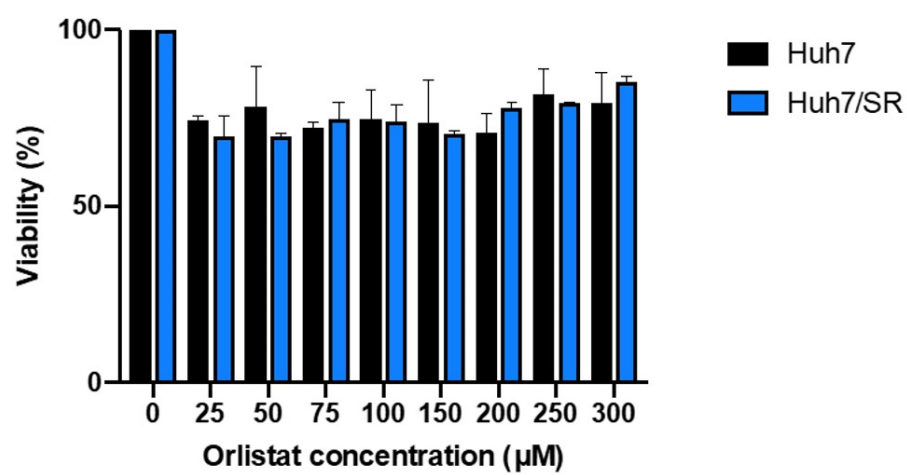

**Figure S1.** Cytotoxicity of orlistat in Huh7 and Huh7/SR cells.

Supplement: Supplementary file 1 [file ijms-23-06501-s001.zip › ijms-1720002-supplementary.pdf]
